# Genetic mapping of escalated aggression in wild-derived mouse strain MSM/Ms: association with serotonin-related genes

**DOI:** 10.3389/fnins.2014.00156

**Published:** 2014-06-11

**Authors:** Aki Takahashi, Toshihiko Shiroishi, Tsuyoshi Koide

**Affiliations:** ^1^Mouse Genomics Resource Laboratory, National Institute of Genetics (NIG)Mishima, Japan; ^2^Department of Genetics, SOKENDAIMishima, Japan; ^3^Mammalian Genetics Laboratory, National Institute of Genetics (NIG)Mishima, Japan

**Keywords:** escalated aggression, wild mouse, MSM/Ms (MSM), genetic mapping, quantitative trait loci (QTLs), chromosome, tryptophan hydroxylase 2 (*Tph2*)

## Abstract

The Japanese wild-derived mouse strain MSM/Ms (MSM) retains a wide range of traits related to behavioral wildness, including high levels of emotionality and avoidance of humans. In this study, we observed that MSM showed a markedly higher level of aggression than the standard laboratory strain C57BL/6J. Whereas almost all MSM males showed high frequencies of attack bites and pursuit in the resident-intruder test, only a few C57BL/6J males showed aggressive behaviors, with these behaviors observed at only a low frequency. Sexually mature MSM males in their home cages killed their littermates, or sometimes female pair-mates. To study the genetic and neurobiological mechanisms that underlie the escalated aggression observed in MSM mice, we analyzed reciprocal F1 crosses and five consomic strains of MSM (Chr 4, 13, 15, X and Y) against the background of C57BL/6J. We identified two chromosomes, Chr 4 and Chr 15, which were involved in the heightened aggression observed in MSM. These chromosomes had different effects on aggression: whereas MSM Chr 15 increased agitation and initiation of aggressive events, MSM Chr 4 induced a maladaptive level of aggressive behavior. Expression analysis of mRNAs of serotonin receptors, serotonin transporter and *Tph2*, an enzyme involved in serotonin synthesis in seven brain areas, indicated several differences among MSM, C57BL/6J, and their consomic strains. We found that *Tph2* expression in the midbrain was increased in the Chr 4 consomic strain, as well as in MSM, and that there was a strong positive genetic correlation between aggressive behavior and *Tph2* expression at the mRNA level. Therefore, it is possible that increased expression of the *Tph2* gene is related to escalated aggression observed in MSM.

## Introduction

Aggression is one of the most conserved behavioral traits in the animal kingdom. It is observed in insects, fish, crustaceans, reptiles, amphibians, birds, and mammals, including humans. However, there are also large differences in the level of aggression between individuals from the same species. These differences can be caused by both environmental and genetic factors. Mouse strains can differ substantially in their levels of aggressive behavior (Ginsberg and Allee, 1942; Scott, 1942), and selective breeding on a certain aspect of aggressive behavior has successfully produced strains of mice that exhibit high and low levels of aggression (Lagerspetz, 1964; Ebert and Hyde, 1976; van Oortmerssen and Bakker, 1981; Gariepy et al., 1996; Sandnabba, 1996). The identification of several knockout mice that show either increased or decreased aggressive behaviors (for reviews, see Miczek et al., 2001; Nelson and Chiavegatto, 2001; Takahashi et al., 2012) indicates that many genes affect aggression. Genetic mapping that involves quantitative trait locus (QTL) analysis has been used to understand the genetic mechanisms that produce the diversity of aggression in natural populations. Four studies have identified genetic loci related to inter-male aggressive behavior in mice (Brodkin et al., 2002; Roubertoux et al., 2005; Nehrenberg et al., 2010; Dow et al., 2011). Comparative analysis of two substrains of BALB/c, which exhibit different levels of aggression, identified variations in the copy number of several sections of DNA between these substrains (Velez et al., 2010). However, identification of the genes or genetic mechanisms that are involved in the individual differences in aggression remains challenging.

The neurobiological mechanisms that control aggression are widely conserved, and the involvement of the serotonin (5-HT) system in aggressive behavior has been confirmed for species from fly to human (for review, see Olivier et al., 1995; Miczek et al., 2007; Yanowitch and Coccaro, 2011). Therefore, it is likely that the 5-HT system is one of the most important endophenotypes for escalated aggression. However, there are numerous receptors for 5-HT, and the effects triggered by their activation can be complex. Pharmacological studies have shown that each receptor type differs in its involvement in aggressive behavior, and that its effect can also vary depending on the brain region (for review, see Takahashi et al., 2010a). Expression analysis of animals that showed escalated aggression after either alcohol consumption or steroid treatment indicated altered expression of some 5-HT receptors specifically in certain brain areas, such as the prefrontal cortex and amygdala (Ambar and Chiavegatto, 2009; Chiavegatto et al., 2010). Thus, it is necessary to examine which receptor type in which brain area is responsible for the individual differences in aggressive behavior.

It has been unclear whether results from studies of laboratory mice are representative of their wild conspecifics. For example, it has been shown that the level of emotionality is attenuated and behavioral patterns are changed in laboratory strains compared with those in wild mice (Holmes et al., 2000; Furuse et al., 2002; Fernandes et al., 2004; Takahashi et al., 2006). The aggressive behavior of wild rodents is also more intense and more diversified than that of laboratory rodents (de Boer et al., 2003). In this study, we examined aggressive behavior in a wild-derived strain of mice, MSM/Ms, and compared it with that of a commonly used laboratory strain, C57BL/6J (B6). MSM originated from Japanese wild mice (*Mus musculus molossinus*) that were captured in 1978; they have been subjected to brother-sister mating and established as an inbred strain (Moriwaki et al., 2009). Behavioral analysis has shown that MSM retains a wide range of behavioral wildness (Koide et al., 2000; Takahashi et al., 2006; Goto et al., 2013). Given the availability of the complete genome sequence of MSM (Takada et al., 2013) and a panel of consomic strains of MSM and B6 (Takada et al., 2008), we considered MSM to be a good model for genetic analysis of aggressive behavior.

In this study, we aimed to identify (1) the genetic basis of escalated aggressive behavior and (2) the involvement of the 5-HT system in the escalated aggression of MSM. For the genetic analysis, we first characterized the aggressive behavior of MSM in comparison with that of B6 in a standard test for territorial aggression (resident-intruder test) and in the daily housing condition. Then, we analyzed a selected set of consomic strains of MSM against a background of B6 to identify the chromosomes that are involved in the escalated aggression of MSM. To examine the involvement of the 5-HT system as one of the intermediate phenotypes (endophenotypes) of the individual differences in aggression, we also examined the mRNA expression of genes for the receptors, synthesizing enzyme and transporter of 5-HT in several brain areas of the consomic strains, MSM and B6.

## Materials and methods

### Subjects and housing

The MSM/Ms (MSM) strain was established and bred at the National Institute of Genetics (NIG). C57BL/6JJcl (B6) mice were purchased from CLEA Japan and bred at NIG. For F1 analysis, we made reciprocal crosses of B6 and MSM (3–4 pairs for each line) to make (B×M)F1 progeny (MSM father) and (M×B)F1 (MSM mother) progeny at NIG. A panel of B6-ChrNMSM consomic strains were established and has been maintained at NIG. The process used to establish this panel was described previously (Takada et al., 2008; Takada and Shiroishi, 2012); briefly, MSM was backcrossed into B6 over 10 generations, and all consomic strains have the same genetic background as B6 except for one pair of chromosomes, which have been substituted for the corresponding MSM chromosome.

Each resident male was housed in pairs with a female of the same strain in transparent polycarbonate cage (22 × 32 × 13.5 cm) with wood chips as bedding material. Intruder males were group-housed at 3–6 per cage in the absence of females. All animals were maintained at NIG with controlled humidity and temperature (50 ± 10%, 23 ± 2°C) under a 12/12-h light/dark cycle (lights on at 6:00 AM). Food and water were freely available. All of the behavioral testing was conducted during the dark period of the photo-cycle (from 6:00 PM to 10:00 PM). All procedures were approved (permit numbers 23-10, 24-10 and 25-10) by the Institutional Committee for Animal Care and Use of the NIG.

### Breeding records

To follow the aggression of MSM and B6 strains in the rearing conditions, we examined their breeding records in the NIG for the previous six years. These records contain information on all the animals from after they were weaned from their parents (at about 3–4 weeks old) until they were used for other studies (at 9–10 weeks old) or used for breeding to produce the next generation. Animals that had been severely injured (lost their tails or had some signs of wounding) or died from attacks by a littermate were recorded as having been subjected to “injurious aggression.” Animals with severe injuries were euthanized once we found evidence of injurious aggression, given that such injuries often result in death within a few days. Given that these records were limited to only the animals in the maintenance colony, we could not follow the animals after they were used for other studies (after 9–10 weeks old). Therefore, there is a limitation in these breeding records insofar as there is the possibility of overlooking incidents of aggression that occur later in the life of these animals.

### Resident-intruder test

Resident males at the age of 7 weeks were housed in pairs with females of the same strain to enhance territorial aggression. In the case of consomic strains, B6 females were sometimes used as the pair-mate if females of the same genotype were not available. After 3 weeks of being housed with a female, the residents were studied for their territorial aggression to an intruder male by using the resident-intruder test. Animals were 10 weeks of age when their aggression was assessed (10–12 weeks in the analysis of consomic strains). Males of a different litter but the same strain were used as the intruders to estimate the aggression in B6 and MSM strains. For reciprocal F1s and consomic strains, we used B6 males as the intruders. The female and pups were removed, and an intruder male was introduced into the home cage of the resident male. Their behaviors were observed for 5 min after the first attack bite, or the intruder was removed after 5 min if no attack occurred. This encounter occurred twice, with a 48-h interval. All behaviors of the animals during the test were videotaped for subsequent behavioral analysis. During the video analysis, the frequency of attack bites and the durations of sideways threats, tail rattles, pursuit, and non-aggressive behaviors (walking, rearing, self-grooming and contact) were quantified as operationally defined and illustrated previously (Grant and Mackintosh, 1963; Miczek and O'Donnell, 1978). The occurrence and duration of those behaviors were recorded by a trained observer using free software established by Akira Tanave (TanaMove0.07, http://www.nig.ac.jp/labs/MGRL/tanaMove.html).

### Quantification of mRNA expression in each brain area

#### Total RNA isolation and cDNA synthesis

Animals were euthanized by CO_2_ inhalation, and their brains were rapidly removed and placed on ice. Seven brain areas (olfactory bulb, prefrontal cortex, striatum, hippocampus, hypothalamus, midbrain, and cerebellum) were dissected by a surgical knife on ice. Briefly, the olfactory bulb was first dissected at the rostral tip of the prefrontal cortex, then the brain was inverted upside-down and the hypothalamus—defined as the area between optic chiasm and mammillary body—was dissected. Next, the midbrain and the cerebellum were obtained. The midbrain area was defined as a coronal section that includes both the superior and the inferior colliculus, and thus both the dorsal raphe and the median raphe nuclei were included in this area. Finally, the brain was sagittally split by the midline, and the prefrontal cortex was dissected from both hemispheres by cutting the 1 mm rostral tip of the frontal cortex at approximately a 45° angle. The whole hippocampal structure was also taken out from both hemispheres, and the striatum was dissected using scissors. These samples were homogenized on ice in Trizol (Invitrogen, USA). Total RNA was extracted and the quantity and quality were checked using a spectrophotometer (NanoDrop, USA). The RNA purity was assessed by determining the OD ratio (260/280 nm > 2) and the 28S/18S rRNA ratio by denaturing RNAs and separating them in a 1% agarose gel with ethidium bromide staining. After DNase treatment (TURBO DNA-free™ kit, Ambion, USA), cDNA was synthesized from each brain area using Primescript Reverse Transcriptase (TaKaRa Bio, Japan). All cDNA samples were stored at −20°C until analysis by real-time PCR.

#### Real-time PCR

The primers used in this study are listed in Table S1. Whereas some primers were chosen by referring to previous work (Chiavegatto et al., 2010), others were selected from the open-access website Primer 3 (v. 0.4.0). Given the extensive polymorphism between B6 and MSM (0.82%), we checked the genome database of MSM (http://molossinus.lab.nig.ac.jp/msmdb/index.jsp) to select primers that were not specific to regions with polymorphisms between B6 and MSM. The expression level of mRNA transcript was quantified using a Thermal Cycler Dice® Real Time System (TP800, TaKaRa Bio, Japan) using SYBR Premix Ex Taq II, Perfect Real Time (TaKaRa Bio, Japan). We used the second derivative maximum (SDM) method to quantifying the expression level of mRNA.

Eight to fifteen animals in each strain at around 11–12 weeks of age were used for this analysis. Each male was housed with a female for 3 weeks and then experienced two aggressive encounters separated by a 48-h interval. Their brains were removed five days after the last aggressive encounter.

#### HPLC measurement of brain 5-HT contents

The midbrain and prefrontal cortex were sampled from males of B6 (*n* = 7), consomic strains of Chr 4 (*n* = 8) and Chr 15 (*n* = 6) that have experiences of about 3 weeks of pair-housing with a female. Animals were euthanized by CO_2_ inhalation, and their brains were rapidly removed, dissected on ice, and frozen at −80°C. Then, tissue samples were weighed and homogenized in 20 μl/mg of ice-cold buffer (0.2 M perchloric acid and 100 μM EDTA-2Na). Samples were centrifuged at 20,000 g for 15 min at 0°C. Supernatants were collected, and the pH was adjusted to be pH 0.3 by adding sodium acetate. Supernatants were filtered through 0.45 μm pore size Cosmonice Filter (Nakalai tesque, Kyoto, Japan) and immediately frozen and stored at −80°C until analysis.

Samples were measured using a high performance liquid chromatography (HPLC) system equipped with an electrochemical detector (ECD-300, Eicom Co., Kyoto, Japan) and ODS column [EICOMPAC PP-ODS II (4.6 × 30 mm) at 25°C (Eicom Co.)]. To measure 5-HT, a mobile phase with 100 mM PBS (pH 5.4), 500 mg/L sodium n-Dodecyl Sulfate (SDS), 13.4 uM EDTA-2Na and 2% methanol in HPLC grade water was used. Samples were diluted by 10%, and 10 μl samples were injected into the HPLC.

### Statistical analysis

Fisher's exact test was used to compare the proportion of animals that showed aggressive behaviors during the 5-min encounter in B6 with those in MSM, F1s, and consomic strains. A repeated-measures Two-Way ANOVA was performed to examine the strain difference in aggressive and non-aggressive behaviors over the two encounters. For the analysis of consomic strains, One-Way ANOVA was conducted using the average value of the first and second encounters owing to the low occurrence of aggressive behavior in the consomic strains. One-Way ANOVA was performed to examine strain differences in the expression of mRNA. When a significant *F* value was obtained, the Tukey-Kramer test and Dunnett's test were conducted as *post-hoc* tests for F1 analysis and consomic analysis, respectively (α = 0.05). For genetic correlation analysis, Pearson's correlations were calculated using the mean score for each strain in all consomic strains and B6. For brain 5-HT contents analysis, outliers that were defined as having datapoints greater than 2 standard deviations away from the mean were excluded from statistical analysis. *T*-test with Bonferroni correction was conducted to compare strain difference of 5-HT contents between B6 and consomic strains.

## Results

### Breeding records of MSM

Although the records kept during the breeding of the MSM strain are incomplete (see Materials and Methods), we found an interesting trend in the differences between strains in terms of their aggression toward same-sex littermates in the home cage. As mentioned above, animals that had been severely injured or died after an attack by another littermate were recorded as having suffered from “injurious aggression.” From the records of MSM, injurious aggression was observed in 13.6% of the housing cages (24 out of 177 cages) that contained multiple male littermates (on average, three males per cage). This injurious aggression was observed after the age of 7 weeks old, when the males are sexually mature. In contrast, injurious aggression was never noted in any of the 265 cages that housed B6 animals. In addition, none of the females of either the MSM or B6 strains showed injurious aggression toward their same-sex cage mates. However, MSM males sometimes attacked their female pair-mates. Females in 9 out of 62 breeding pairs of MSM (14.5%) were injured or killed.

### Resident-intruder test of MSM

**Figure 1:**
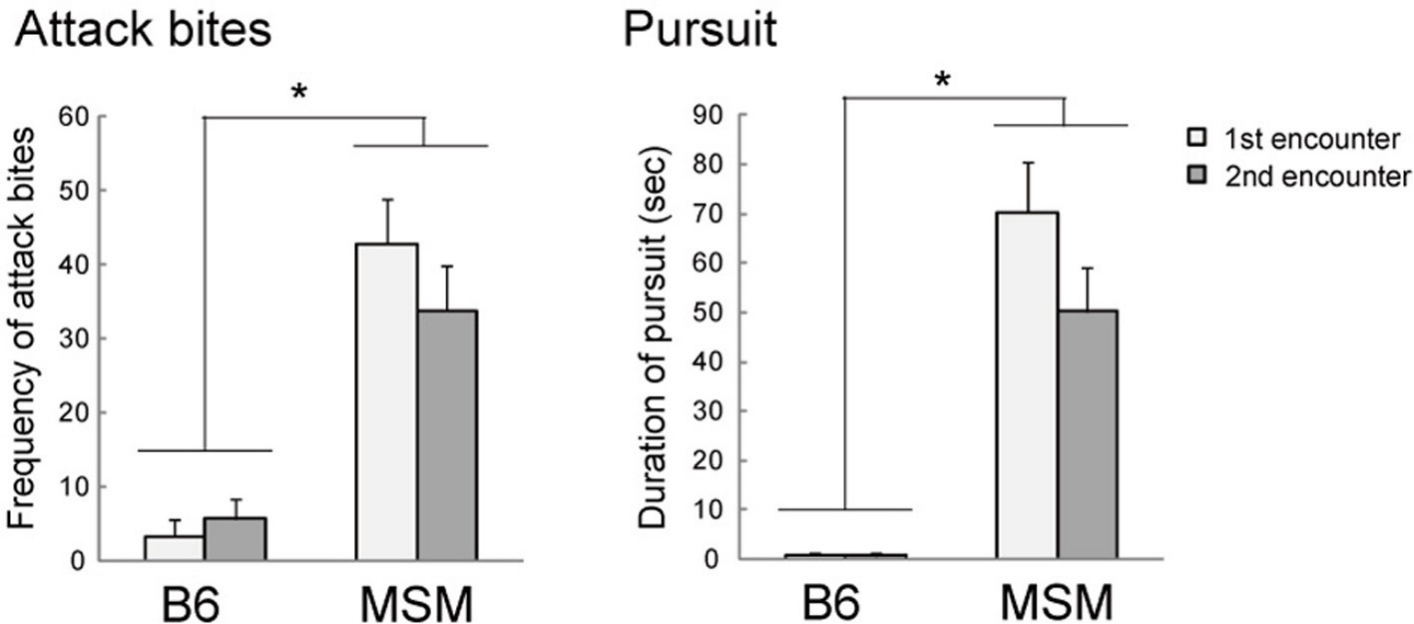
**Aggressive behaviors of MSM and B6 in the resident-intruder test**. MSM showed significantly more attack bites and pursuit than B6 at both first and second encounters. ^*^Significant strain difference between B6 and MSM (*p* < 0.05).

Mice of the MSM strain showed higher levels of inter-male aggression than their B6 counterparts in the resident-intruder test. Whereas 14 resident males out of 16 pairs (87.5%) of MSM showed attack bites at the first encounter, only 2 residents out of 19 pairs (10.5%) of B6 showed aggressive behaviors (Table 1). Fisher's exact test showed that the number of animals that showed aggressive behavior was significantly higher in MSM than in B6 during both first and second encounters. We then analyzed the detailed behaviors during the 5-min encounter from the video recordings. Repeated-measures Two-Way ANOVA showed significant strain differences in aggressive behaviors, including attack bites, pursuit and attack latency [*F*_(1, 33)_ > 43.456, *p* < 0.0001], as well as non-aggressive behaviors including walking, rearing and contact [*F*_(1, 33)_ = 4.847, *p* < 0.035] (Table 1). Compared with B6 mice, MSM mice showed a significantly higher frequency of attack bites and longer pursuit (Figure 1), as well as shorter attack latency. In contrast, B6 showed more non-aggressive behaviors (walking, rearing and contact) than MSM. A significant strain × encounter interaction was observed only for walking [*F*_(1, 33)_ = 4.247, *p* = 0.0473] and B6 showed a significant decrease of walking in the second encounter compared with that in the first encounter, but there was no change in MSM.

**Table 1:** **Resident-intruder test in MSM and B6 males**.

	**B6**		**MSM**	
	**1st**	**2nd**	**1st**	**2nd**
**NUMBER OF ANIMALS**				
Total resident males	19	19	16	16
Males that showed attack bites	2	4	14^+^	15^+^
% aggressive males	10.5 %	21.1 %	87.5 %	93.8 %
**DETAILED BEHAVIORS**				
Attack bites (f)	3.4 ± 2.3	5.7 ± 2.7	42.9 ± 6.0^**^	33.8 ± 6.1^**^
Sideways threats (d)	2.9 ± 2.0	5.7 ± 4.0	6.2 ± 1.7	4.0 ± 0.9
Tail rattles (d)	1.2 ± 0.8	2.5 ± 1.4	8.8 ± 1.4^*^	8.1 ± 3.5
Pursuit (d)	0.8 ± 0.7	0.7 ± 0.5	70.3 ± 10.2^**^	50.1 ± 8.9^**^
Walking (d)	124.8 ± 8.8	102.5 ± 7.4	57.4 ± 5.8^**^	62.1 ± 8.0^**^
Rearing (d)	43.1 ± 3.9	34.6 ± 4.6	18.5 ± 3.9^**^	18.7 ± 4.1^*^
Grooming (d)	7.8 ± 1.8	8.6 ± 2.0	6.3 ± 3.7	9.3 ± 4.9
Contact (d)	44.7 ± 5.5	39.6 ± 6.3	21.7 ± 12.7	11.9 ± 10.1^*^
Attack latency	287.9 ± 9	263.5 ± 18	172.7 ± 23^**^	99.2 ± 24^**^

Significant strain differences between B6 and MSM are indicated as

+ p < 0.05 by Fisher's exact test, and

* p < 0.05 and

** *p < 0.01 by the Tukey-Kramer test. (f): frequency, (d): duration*.

**Figure 2:**
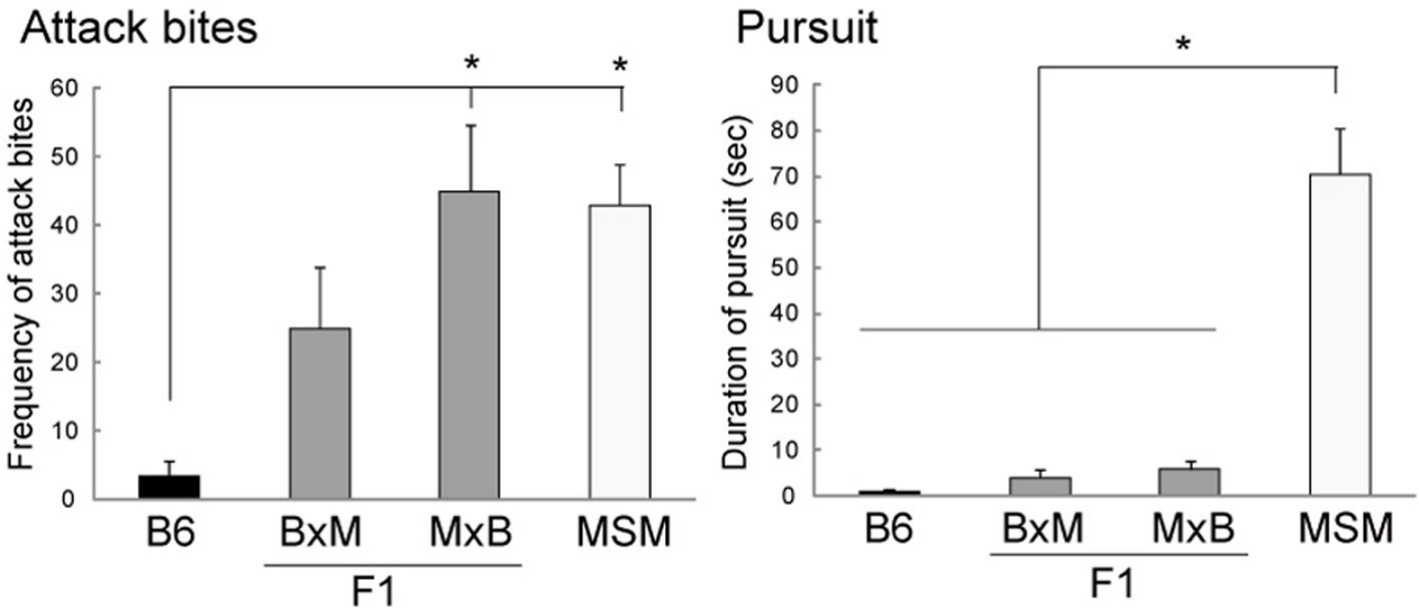
**Aggressive behaviors of reciprocal F1 crosses between B6 and MSM in the resident-intruder test**. Whereas B×M represents F1 males that have a B6 mother, M×B represents those with an MSM mother. ^*^Significant strain difference (*p* < 0.05).

Aggressive behaviors of the reciprocal F1 heterozygotes, (B×M)F1 and (M×B)F1, were also examined and compared with those of their parental strains, B6 and MSM (Figure 2). Males of (M×B)F1, which have MSM as a mother, showed high territorial aggression similar to that of MSM in terms of the proportion of aggressive males, the frequency of attack bites, the duration of tail rattles, and short attack latency. In contrast, the males of (B×M)F1, which have MSM as a father, showed an intermediate level of aggression between B6 and MSM in these indices (Figure 2 left, Table 2). On the other hand, the frequency of pursuit (a characteristic behavior of MSM) in both (B×M)F1 and (M×B)F1 was similar to the level of B6 at both first and second encounters (Figure 2 right). Furthermore, breeding records showed that there was no injurious aggression in either F1 groups during daily housing condition.

**Table 2:** **Resident-intruder test in reciprocal F1 crosses**.

	**(B × M)F1**		**(M × B)F1**	
	**1st**	**2nd**	**1st**	**2nd**
**NUMBER OF ANIMALS**				
Total resident males	10	10	10	10
Males that showed attack bites	6	8	9^a^	10^a^
% aggressive males	60 %	80 %	90 %	100 %
**DETAILED BEHAVIORS**				
Attack bites (f)	25.0 ± 8.9	31.6 ± 7.4^a^	44.9 ± 9.7^a^	42.8 ± 11.5^a^
Sideways threats (d)	8.2 ± 2.3	8.3 ± 1.6	13.5 ± 2.8^a^	11.4 ± 3.3
Tail rattles (d)	4.6 ± 1.6	12.3 ± 4.7	17.0 ± 6.1^a^	19.8 ± 7.5^a^
Pursuit (d)	4.0 ± 1.8^b^	7.6 ± 3.4^b^	5.9 ± 1.9^b^	4.4 ± 1.7^b^
Walking (d)	79.3 ± 5.2^a^	80.5 ± 7.0	73.5 ± 7.2^a^	55.7 ± 8.8^a^
Rearing (d)	50.3 ± 10.1^b^	36.4 ± 6.8	32.7 ± 7.5	16.7 ± 6.7
Grooming (d)	13.5 ± 5.5	9.8 ± 2.0	9.3 ± 4.8	9.5 ± 3.0
Contact (d)	48.1 ± 15.4	26.9 ± 11.0	43.0 ± 13.8	8.4 ± 5.8
Attack latency	197.0 ± 27^a^	111.9 ± 33^a^	89.4 ± 26^a^^b^^c^	58.6 ± 14^a^

Significant differences compared with B6 (

a ), MSM (

b ) (Table 1) and (B×M)F1 (

c ) by Tukey-Kramer test (p < 0.05). (f): frequency, (d): duration.

### Analysis of B6/MSM consomic strains

This study examined five strains (that correspond to chromosomes Chr 4, Chr 13, Chr 15, Chr X, and Chr Y) of twenty-nine consomic strains. We chose these strains in this analysis because a previous study that used the social interaction test indicated that a subset of male pairs in the consomic strains of Chr 4, 13, 15, and 17 showed attack bites during the test, whereas the other strains did not show any aggressive behavior (Takahashi et al., 2010c). In addition, we examined consomic strains of Chr X (XT, which has the telomeric half of Chr X from MSM) and Chr Y because some reports have mentioned the role of sex chromosomes in aggressive behavior (Selmanoff et al., 1975; Sluyter et al., 1996; Brodkin et al., 2002; Roubertoux et al., 2005). Unfortunately, Chr 17 and Chr XC (centromeric half of Chr X from MSM) consomic strains were not included in the analysis because we could not obtain a sufficient number of animals.

**Figure 3:**
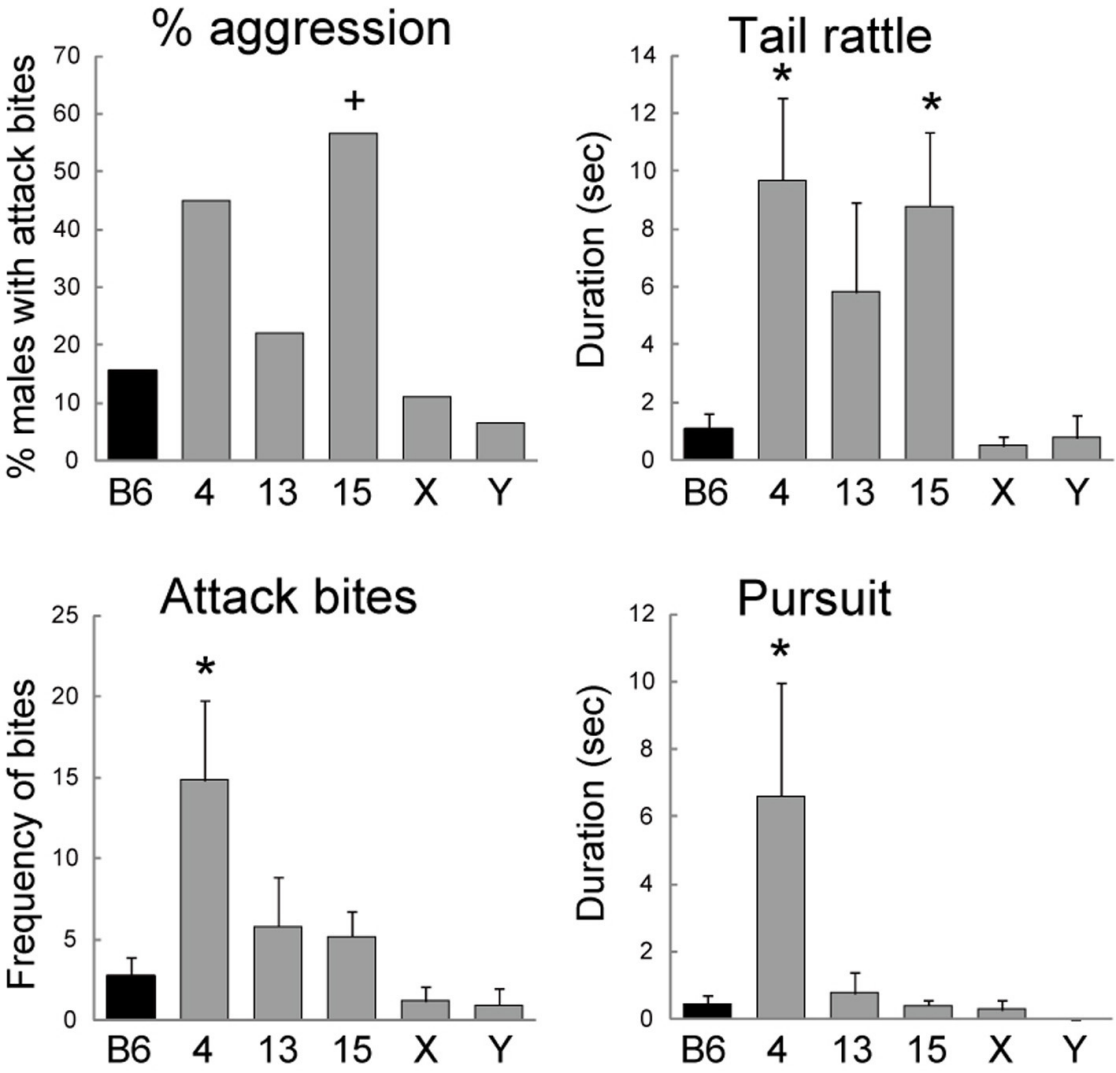
**Aggressive behaviors of consomic strains of MSM in the resident-intruder test**. The numbers (4, 13, 15) and letters (X, Y) indicate the chromosome that was substituted for that of MSM in the B6 genetic background. Significant difference compared with B6 by Dunnett's test (^*^p < 0.05) or Fisher's exact test (^+^*p* < 0.05).

All of the consomic strains analyzed in this study showed a low level of aggressive behavior similar to that of B6 at the first encounter (Table 3). By contrast, we found that the consomic strain of Chr 15 showed a higher level of initiation of aggressive behavior than B6 at the second encounter. Fisher's exact test indicated that the proportion of animals that showed attack bites was significantly higher in the Chr 15 strain than in B6 (*p* = 0.0317, Figure 3). We then analyzed the detailed behaviors during the 5-min encounter. One-Way ANOVA revealed a significant main effect of strain in attack bites [*F*_(5, 121)_ = 4.081, *p* = 0.0019], tail rattles [*F*_(5, 121)_ = 4.381, *p* = 0.0011], sideways threats [*F*_(5, 121)_ = 2.357, *p* = 0.0443], and pursuit [*F*_(5, 121)_ = 3.495, *p* = 0.0055]. A *post-hoc* Dunnett's *t*-test showed that, compared with B6, the consomic strain of Chr 4 exhibited significantly higher levels of attack bites, tail rattles, sideways threats, and pursuit (Figure 3). In addition, the Chr 15 consomic strain showed a significantly higher level of tail rattles than B6 (Figure 3). In terms of non-aggressive behaviors, a significant main effect of strain was observed only for walking [*F*_(5, 121)_ = 8.981, *p* < 0.0001], and the Chr X consomic strain showed more walking than B6.

**Table 3:** **Resident-intruder test in five consomic mouse strains and B6**.

	**B6**	**Chr 4**	**Chr 13**	**Chr 15**	**Chr X**	**Chr Y**
**NUMBER OF ANIMALS**						
Total resident males	32	20	18	23	18	15
Males that showed attack bites	5 (2)	9 (5)	4 (1)	13 (2)^+^	2 (0)	1 (0)
% aggressive males	15.6 %	45.0 %	22.2 %	56.5 %	11.1 %	6.7 %
**DETAILED BEHAVIORS**						
Attack bites (f)	2.7 ± 1.2	14.8 ± 5.0^*^	5.8 ± 3.1	5.2 ± 1.5	1.2 ± 0.8	1.0 ± 1.0
Sideways threat (d)	2.6 ± 1.4	7.8 ± 2.4^*^	3.3 ± 1.9	4.1 ± 1.3	1.2 ± 0.9	0.6 ± 0.6
Tail rattles (d)	1.1 ± 0.5	9.7 ± 2.8^*^	5.8 ± 3.1	8.8 ± 2.5^*^	0.5 ± 0.3	0.8 ± 0.8
Pursuit (d)	0.4 ± 0.3	6.6 ± 3.4^*^	0.8 ± 0.6	0.4 ± 0.2	0.3 ± 0.3	0.0 ± 0.0
Walking (d)	108.0 ± 4.7	100.7 ± 6.6	123.1 ± 6.6	92.7 ± 4.1	140.9 ± 4.0^*^	106.1 ± 7.9
Rearing (d)	39.7 ± 2.4	37.4 ± 3.2	41.5 ± 3.2	37.6 ± 2.5	36.8 ± 2.2	37.1 ± 2.9
Grooming (d)	7.6 ± 0.9	9.1 ± 2.4	5.9 ± 0.8	11.0 ± 1.5	5.5 ± 1.0	6.4 ± 1.1
Contact (d)	44.1 ± 3.6	42.0 ± 5.8	43.0 ± 6.0	35.3 ± 3.6	32.5 ± 3.0	28.4 ± 4.8
Attack latency	278.3 ± 11.0	189.6 ± 27.1^*^	262.9 ± 20.3	219.8 ± 19.1^*^	276.2 ± 16.6	288.3 ± 11.7

Significant strain difference between B6 and MSM by Dunnett's t-test (

* p < 0.05) or Fisher's exact test (

+ *p < *0.05*), (f): frequency, (d): duration. Numbers in parentheses are the numbers of males that showed attack bites at the first encounter. For the detailed behaviors, the averages of first and second encounters are indicated. For attack latency, the result of the second encounter is indicated*.

The escalation of aggression in the Chr 4 consomic strain was also observed in the daily housing condition according to the breeding record. During the 3 weeks of housing with a female before the test, we also checked the occurrence of injurious aggression toward a female pair-mate. The Chr 4 consomic strain showed injurious aggression toward females, and females in 8 out of 20 pairs were injured. This strain also showed injurious aggression toward male cage mates (11.9%, 15 out of 126 cages). On the other hand, we did not observe any cages with injurious aggression in the other consomic strains of Chr 13, Chr 15, Chr X, and Chr Y.

### Strain difference in the mRNA expression of 5-HT receptors, enzyme and transporter

**Figure 4:**
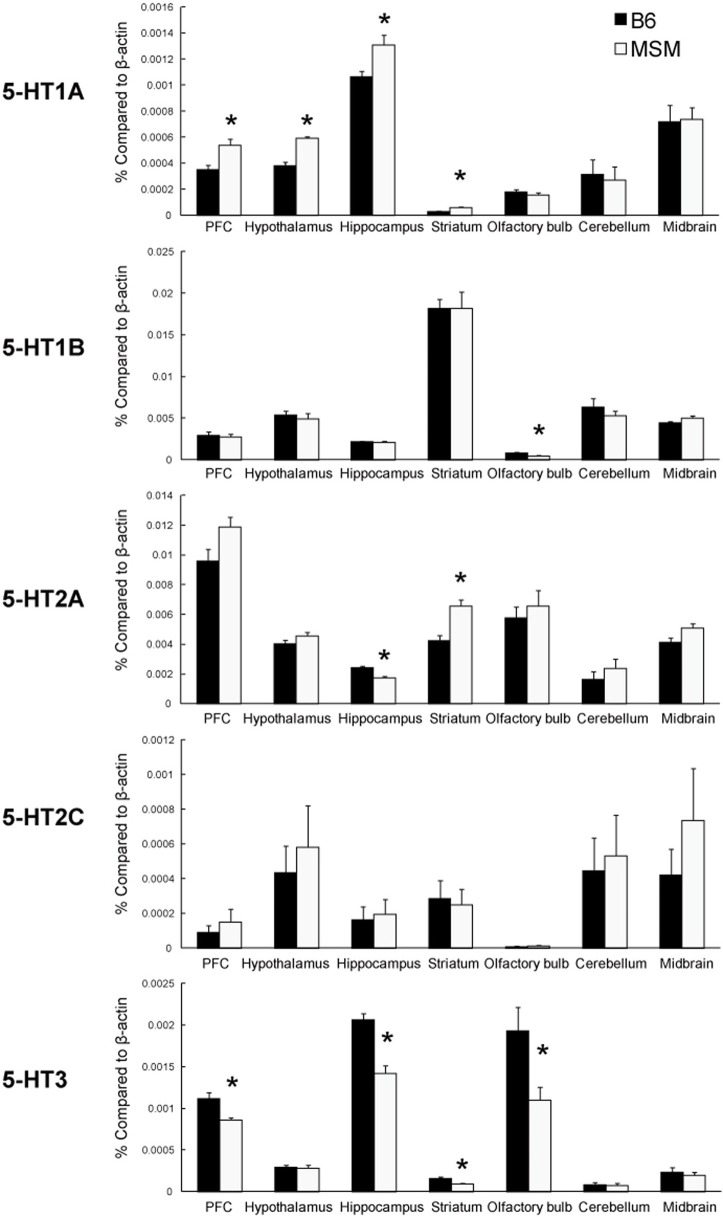
**Real-time quantitative PCR analysis of the mRNA expression of 5-HT receptors in seven brain areas of MSM and B6**. ^*^Significant strain difference between B6 and MSM (*p* < 0.05).

**Figure 5:**
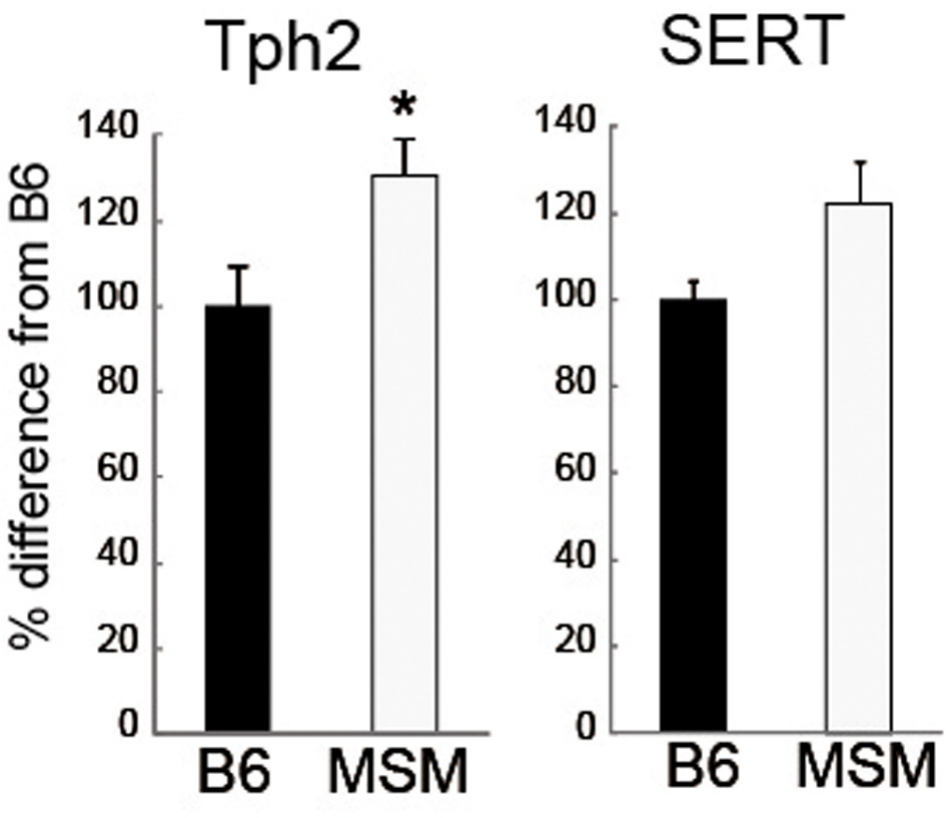
**mRNA expressions of tryptophan hydroxylase 2 (*Tph2*) and 5-HT transporter (*SERT*) in the midbrain of MSM and B6**. ^*^Significant strain difference between B6 and MSM (*p* < 0.05).

To evaluate the difference in the 5-HT system between B6 and MSM, we examined the expression level of 5-HT receptor mRNAs in seven brain areas of B6 and MSM using quantitative real-time PCR (Figure 4). The mRNA expression of 5-HT_1A_ receptor was significantly higher in MSM than in B6 in the prefrontal cortex, hypothalamus, hippocampus, and striatum [*F*_(1, 11)_ > 6.043, *p* ≤ 0.0318], whereas there was no difference between the strains in levels of the same transcript in the olfactory bulb, cerebellum and midbrain. For 5-HT_1B_ mRNA, a significant difference in expression was observed only in olfactory bulb [*F*_(1, 11)_ = 7.208, *p* = 0.0199], with no differences observed in the six other areas tested. The expression difference in 5-HT_2A_ receptor mRNA was bidirectional: MSM showed lower 5-HT_2A_ mRNA in hippocampus [*F*_(1, 11)_ = 20.235, *p* = 0.0009], but a higher level in striatum [*F*_(1, 11)_ = 17.120, *p* = 0.0017], compared with B6. There was no significant strain difference in the expression of 5-HT_2C_ mRNA. On the other hand, 5-HT_3A_ mRNA expression was significantly lower in MSM than in B6 in the prefrontal cortex, hippocampus, striatum and olfactory bulb [*F*_(1, 11)_ > 6.133, *p* ≤ 0.0308]. We also examined the mRNA expression of serotonin transporter (SERT) and Tph2 in the midbrain area (Figure 5). The Tph2 expression was significantly higher in MSM than in B6 [*F*_(1, 14)_ = 10.901, *p* = 0.0052]. The SERT expression was also higher in MSM, but the difference was not statistically significant [*F*_(1, 14)_ = 3.770, *p* = 0.0726].

**Figure 6:**
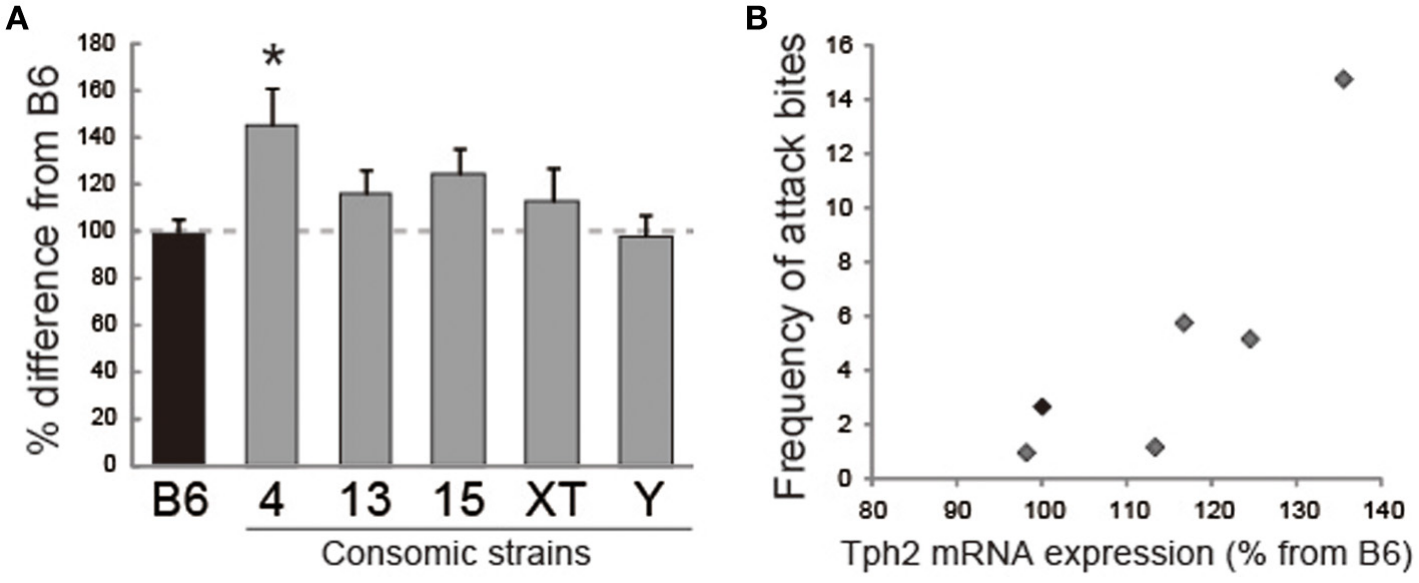
**Analysis of the abundances of *Tph2* transcripts in five consomic strains and B6**. **(A)** Chr 4 consomic strains showed a significant increase of *Tph2* mRNA expression in the midbrain. ^*^Significant difference compared with B6 (*p* < 0.05). **(B)** Positive genetic correlation between *Tph2* expression and the frequency of attack bites.

To examine whether these strain differences observed in the expression of 5-HT-related mRNA correspond to heightened aggression in MSM, we then examined the expression of 5-HT receptors and Tph2 using five consomic strains, and its genetic correlation with aggressive behaviors (Table 4). This expression analysis showed that the Chr 4 consomic strain, which exhibited escalated and injurious aggressive behavior, had significantly increased *Tph2* mRNA expression in the midbrain (Figure 6A). This strain, but not MSM, also showed elevated expression of 5-HT_2A_ receptor in the prefrontal cortex relative to B6. On the other hand, the Chr 15 consomic strain, which showed a higher level of initiation of aggressive behavior than the other strains tested, did not show any significant difference in the expression of 5-HT receptor at the mRNA level. There was also a slight increase (124%) of Tph2 expression in the Chr 15 consomic strain, but this was not statistically significant. Significant positive genetic correlations were shown between *Tph2* mRNA expression and aggressive behaviors (% aggressive animals, bites and tail rattles, *r* ≥ 0.82, *p* < 0.05; Figure 6B). In addition, a positive correlation was observed between 5-HT_2A_ expression in the prefrontal cortex and attack bites (*r* = 0.81, *p* = 0.0527). Although they were not statistically significant, moderate negative correlations were observed between 5-HT_1A_ or 5-HT_3A_ in the prefrontal cortex and aggressive behaviors (Table 4). However, there were no correlations between 5-HT receptor expression in the hippocampus and any aggressive behaviors.

**Table 4:** **The expression of 5-HT receptors and Tph2 mRNAs in five consomic strains and their genetic correlations with aggressive behaviors**.

	**Prefrontal cortex**			**Hippocampus**			**Midbrain**
	**5-HT1A**	**5-HT2A**	**5-HT3A**	**5-HT1A**	**5-HT2A**	**5-HT3a**	**Tph2**
**mRNA EXPRESSION (% DIFFERENCE FROM B6)**							
Chr 4	86 ± 9	150 ± 14^*^	98 ± 11	85 ± 9	97 ± 4	72 ± 10	146 ± 15^*^
Chr 13	108 ± 12	146 ± 27^*^	125 ± 27	164 ± 14^*^	79 ± 6	55 ± 6	117 ± 10
Chr 15	113 ± 16	91 ± 13	84 ± 13	83 ± 9	83 ± 8	68 ± 13	124 ± 11
Chr X	114 ± 12^*^	92 ± 14	126 ± 18	93 ± 13	82 ± 10	39 ± 9^*^	113 ± 14
Chr Y	106 ± 2	86 ± 9	106 ± 26	81 ± 8	99 ± 15	77 ± 11	98 ± 10
**GENETIC CORRELATION**							
% aggressive animals	−0.49	0.31	−0.69	−0.07	−0.21	0.03	0.82^*^
Attack bites	−0.46	0.81^+^	−0.31	0.00	0.12	0.06	0.85^*^
Tail rattles	−0.53	0.61	−0.49	0.15	−0.22	−0.05	0.90^*^
Pursuit	−0.32	0.72	−0.23	−0.20	0.31	0.07	0.74^+^

Significant strain differences between B6 and each consomic strain by Dunnett's t-test (^*^p < 0.05) for mRNA expression. Genetic correlations were calculated by Pearson's correlations.

* *p < 0.05*,

+ *p < 0.10*.

**Figure 7:**
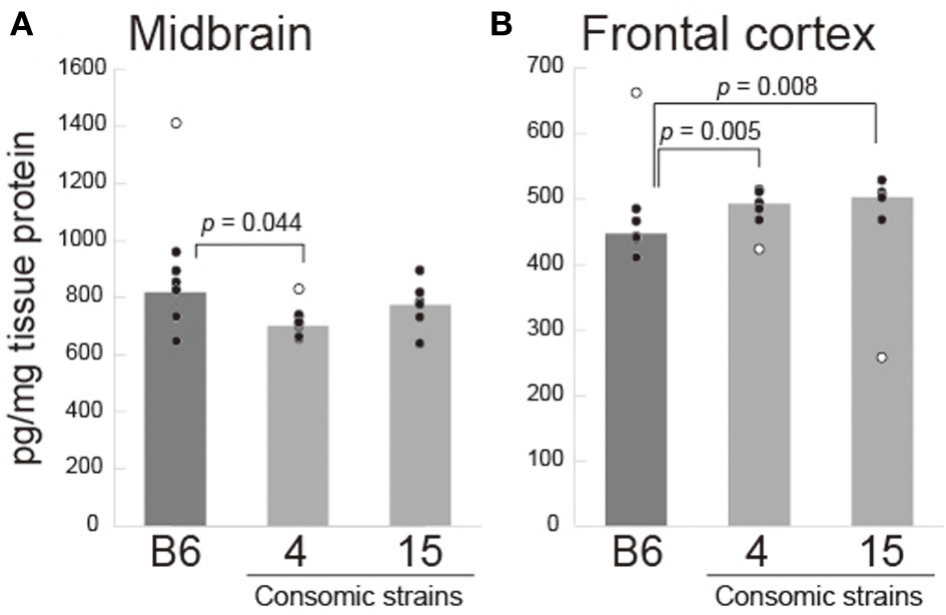
**Brain 5-HT content (pg/mg tissue) in the midbrain (A) and prefrontal cortex (B) in B6 and consomic strains of Chr 4 and Chr 15**. Each circle indicates each individual's 5-HT content, and open circles indicate outliers that were excluded for statistical analysis. The *p*-values were calculated by *t*-test with Bonferroni correction.

To examine whether the increase of *Tph2* expression at the mRNA level can affect the brain 5-HT contents, we measured 5-HT contents in the midbrain and prefrontal cortex in B6, Chr 4, and Chr 15 consomic strains. Unexpectedly, we found that the 5-HT contents were decreased in the midbrain homogenate of Chr 4 consomic strain compared to B6 [*t*_(11)_ = −2.669, *p* = 0.0436; Figure 7A]. There was no change in 5-HT contents in the midbrain sample of Chr 15 consomic strain. By contrast, in the prefrontal cortex, both Chr 4 consomic [*t*_(11)_ = 3.951, *p* = 0.0046] and Chr 15 consomic strains [*t*_(9)_ = 3.820, *p* = 0.0082] showed increases in 5-HT contents compared to B6 (Figure 7B).

## Discussion

### Escalated aggression in MSM

This study revealed that a Japanese wild-derived mouse strain, MSM, has an escalated level of aggressive behavior compared with the commonly used laboratory strain B6. This aggressive behavior of MSM was characterized by frequent pursuit (chasing) behavior, in addition to attack bites. This pursuing contrasts with the behavior of not only B6 strain but other laboratory mouse lines, such as ICR and CFW (Takahashi et al., 2010b). Fierce chasing behavior with no respite was also observed in wild mice (Crowcroft, 1966). It can thus be postulated that MSM retains some patterns of aggressive behavior that are observed in wild mice. From the breeding records of MSM, we found that some MSM males showed a high level of injurious attacks (or killing) against cage mates. This injurious behavior was also directed toward female mates. Therefore, under the laboratory housing conditions, the aggression of male MSM mice appears to be maladaptive because there is a lack of inhibition of aggressive behavior of MSM even toward inappropriate targets (females). The expression of escalated aggressive behavior in MSM was observed after sexual maturation, suggesting that the sex steroids might have an important role in triggering their aggression.

### Genetic analysis of escalated aggressive behavior

The analysis of reciprocal F1s showed that there is a different mode of inheritance for some indices of escalated aggression observed in MSM. Given that we did not observe any injurious aggression and also no increase of pursuit in both F1 intercrosses, these phenotypes are considered as recessive traits. On the other hand, the frequency of attack bites and tail rattles, as well as the percentage of aggressive animals, were higher in the F1 intercrosses than in B6, whereas attack latency was lower in F1 intercrosses than in B6. Thus, these behaviors have either a dominant or an additive mode of inheritance. Interestingly, we found differences between the reciprocal F1 crosses in these phenotypes: whereas (M×B)F1, which has MSM as a mother, showed a pronounced increase of aggression similar to that of MSM, (B×M)F1, which has MSM as a father, showed a level of aggression intermediate between that of B6 and MSM. The genetic differences between (M×B)F1 and (B×M)F1 are only in sex chromosomes and mitochondrial DNA; all autosomes are identically heterozygote. However, our analysis of consomic strains did not find any effect of the sex chromosomes on intermale aggression despite the sex chromosomes previously being implicated in aggressive behaviors by the analysis of both hybrid or congenic strains of Y (Selmanoff et al., 1975; Sluyter et al., 1996) as well as by QTL mapping (Brodkin et al., 2002; Roubertoux et al., 2005). Therefore, it is likely that genetic loci involved in escalated aggression of MSM are not localized on the sex chromosomes, or that they need to interact with other autosomal loci (Maxson et al., 1979) or with the specific maternal environment (Carlier et al., 1991) to exert their behavioral effects. Also, it has been reported that the difference in maternal behavior could change aggressive behavior of same-genotype offspring (Bester-Meredith and Marler, 2001; Cox et al., 2013). Another possible reason for differences between reciprocal F1s is the genomic imprinting, which causes preferential expression of the maternal or paternal allele, and it has reported that more than 1300 loci showed differential allelic expression in mouse brain (Gregg et al., 2010). Whether this maternal effect observed in the reciprocal F1s is due to the maternal behavior or the epigenetic modification in the maternal loci or a complex genetic interaction should be clarified in the future.

Analysis of consomic strains identified two chromosomes, Chr 4 and Chr 15, which are involved in these different aspects of aggressive behavior. Our results indicated that Chr 15 of MSM increased the proportion of animals that initiated aggressive behavior and the frequency of tail rattles. However, the frequencies of attack bites and pursuit were similar to those in B6, and there was no injurious aggression observed in Chr 15 consomic males. These findings suggest that there is genetic locus that increases agitation and the initiation of aggressive behaviors on Chr 15. On the other hand, we found that the consomic strain of Chr 4 showed a maladaptive level of aggression. The breeding records from daily housing conditions indicated that the Chr 4 consomic males showed injurious aggression toward both their same-sex littermates and their female mates. In the resident-intruder test, Chr 4 males showed increased frequencies of attack bite and a longer duration of pursuit. On the other hand, the proportion of animals that showed aggressive behavior was not significantly different from that in B6. This indicated that Chr 4 consomic animals showed exaggerated aggressive behavior after aggression had been triggered. Thus, a genetic locus on MSM Chr 4 might be responsible for the maladaptive aspect of aggression observed in MSM. Our findings indicate that there are different genetic bases for agitation and readily provoked aggressive behavior (Chr 15) and for escalated maladaptive aggressive behavior (Chr 4). A role for Chr 4 in controlling aggression is consistent with a report that strains of A/J and B6, which carry substitutions in Chr 4, also showed severe fighting in the housing cage (Singer et al., 2005). In addition, QTL analysis of the initiation of aggression of F2 mice derived from a cross between BALB/c and A/J strains toward an intruder dangled at a corner of test cage identified an aggression-related QTL on Chr 15 (Dow et al., 2011).

Compared with MSM, all tested consomic strains showed a low level of aggression at the first encounter compared with MSM. This indicates that the genetic effect of either Chr 4 or Chr 15 is not very large and that multiple loci contribute to the escalated aggression of MSM.

### Escalated aggression and the 5-HT system

Expression analysis of several 5-HT receptors, *Tph2* and a serotonin transporter showed several strain differences in mRNA expression between B6 and MSM. To examine which differences in the 5-HT system between B6 and MSM actually correlate with the level of aggressive behavior, we analyzed the mRNA expression in consomic mouse strains and calculated the genetic correlation between mRNA expression and aggressive behavior. The result showed highly positive correlations between the level of the 5-HT synthetic enzyme *Tph2* in the midbrain and several aggressive behaviors. Both MSM and the consomic strain of Chr 4 showed injurious aggression toward both male and female cage mates, and also showed large increase in *Tph2* expression compared with that in B6. The consomic strain of Chr 15 that showed high agitation toward male intruders also showed a modest increase in the abundance of *Tph2* mRNA.

Although Tph2 has been implicated in aggression because it directly affects the activity of 5-HT neurons, the relationship between Tph2 activity and the level of aggression seems to be complex. It has been shown that both male and female *Tph2* gene knockout mice, which have very low levels of 5-HT but normal 5-HT neuron development (Gutknecht et al., 2008), exhibited escalated aggressive behavior in both their daily housing conditions and the resident-intruder test (Alenina et al., 2009; Angoa-Pérez et al., 2012; Mosienko et al., 2012). Knock-in mice with an R439H point mutation in the *Tph2* gene, which causes an 80% reduction of enzymatic activity, consistently showed increased attack behavior compared with the wild type in a neutral test area (Beaulieu et al., 2008). These results consistently indicate that a reduction of Tph2 activity, and hence a reduction in brain 5-HT, corresponds to exaggerated aggressive behavior. On the other hand, strain comparison studies of Tph2 activity have shown a positive correlation between the activity of Tph2 and the level of aggression in several mouse strains (Kulikov and Popova, 1996; Kulikov et al., 2005). A single-nucleotide polymorphism in the gene that encodes Tph2 (C1473G) affects the activity of Tph2 (Zhang et al., 2004; Kulikov et al., 2005; Osipova et al., 2009), and congenic mice that have the C1473G-type locus from the CC57BR strain, which causes low Tph2 activity in the midbrain, showed a reduced level of aggression compared with the parental B6 strain (Osipova et al., 2009). Given these findings, it is possible that deviation of 5-HT function from its appropriate level in either direction may escalate the level of aggression. Our finding that MSM expresses increased levels of the mRNA that encode Tph2 seems to be consistent with the latter findings, namely, a positive correlation between the abundance of *Tph2* mRNA and aggressive behavior. However, when we measured the 5-HT contents in the brain, we found mixed results; Chr 4 consomic strain showed reduced 5-HT in the midbrain but increased 5-HT in the prefrontal cortex. By contrast, Chr 15 consomic strain showed increased 5-HT contents in the prefrontal cortex, but no change in the midbrain. This different pattern of change of 5-HT contents may correspond to the different type of aggression observed in Chr 4 and Chr 15 consomic strains. While it is unclear how these complex effects on 5-HT contents in each brain region were produced by increased *Tph2* mRNA expression, our data suggest that the expression of *Tph2* can be a good candidate for an endophenotype of escalated aggression observed in MSM. Given that MSM has the same genotype at the C1473G locus as B6 (Osipova et al., 2010) and that the *Tph2* gene is also localized on Chr 10, there should be no C1473G-related difference in Tph2 activity between B6 and MSM or the Chr 4 or Chr 15 consomic strain. Further investigation is thus required to examine the direct relationship between Tph2 expression and the increased aggression observed in MSM.

This study identified the involvement of two chromosomes, Chr 4 and Chr 15, in different aspects of escalated aggression in MSM. Our result of a correlation between *Tph2* and aggressive behaviors suggests that a difference in the expression of Tph2 in midbrain can be an endophenotype for the escalated aggression in MSM. The analysis of a panel of congenic strains for either Chr 4 or Chr 15, in which only small segment of chromosome was substituted with MSM, will lead to identify genes that are involved in the escalated aggression and their relationships to the 5-HT system.

### Conflict of interest statement

The authors declare that the research was conducted in the absence of any commercial or financial relationships that could be construed as a potential conflict of interest.
