# Supplementary material for: Genetic mapping of escalated aggression in wild-derived mouse strain MSM/Ms: association with serotonin-related genes
Source: Front Neurosci. 2014 Jun 11;8:156. doi: 10.3389/fnins.2014.00156 (PMC4052355; doi:10.3389/fnins.2014.00156)
Supplement: Supplementary file 1 [file DataSheet1.DOCX]

Supplementary Table 1

| Gene | Forward | Reverse | Size (bp) |
| --- | --- | --- | --- |
| 5-HT_1A_ receptor | caccgatctcatggtgtcag | acatccagggcgataaacag | 111 |
| 5-HT_1B_ receptor | tatcacctgttgcactgctt | gagatggagaagacccacac | 150 |
| 5-HT_2A_ receptor* | agaaccccattcaccatagc | atcctgtagcccgaagactg | 119 |
| 5-HT_2C_ receptor | tgtcagccattgtttcattc | tgacttgagcaaggtttgaa | 103 |
| 5-HT_3A_ receptor* | cttcccctttgatgtgcag | ccactcgccctgatttatg | 139 |
| SERT | tctctgcatcatgctcatct | ggacaatgtaagggaaggtg | 107 |
| Tph2* | tgcaagcaagagggtcaact | catgcttcaattctccgatg | 70 |
| Beta-actin | tgacaggatgcagaaggaga | cgctcaggaggagcaatg | 75 |

* Used by Chiavegatto *et al*. (2010).
